# Using Sina-Weibo microblogs to inform the development and dissemination of health awareness material about Zika virus transmission, China, 2016–17

**DOI:** 10.1371/journal.pone.0261602

**Published:** 2022-01-27

**Authors:** Qian Hou, Yueqiao Zhao, Xiaoge Su, Rong Rong, Sujian Situ, Ying Cui

**Affiliations:** 1 Health Communication Center (Health Hotline Management Office), China Centers for Disease Control and Prevention, Beijing, China; 2 Division of Global Health Protection, United States Centers for Disease Control and Prevention, Beijing, China; Public Health England, UNITED KINGDOM

## Abstract

**Background:**

On 1 February 2016, the World Health Organization declared Zika transmission a public health emergency of international concern. Monitoring and responding to community awareness, concern, and possible knowledge gaps are critical during public health emergencies. Here, we describe the review and analysis of micro-blogs posted on Sina-Weibo, China’s largest social media platform, to develop and disseminate a Zika virus education campaign.

**Methods:**

We used CYYUN Voice Express’ Weibo Spider tool and the search terms of "Zhaika" OR "Zika" OR "Zikv” to capture microblogs about Zika virus retrospectively from February 1 to December 31, 2016 and prospectively from June 1 to November 15, 2017. We described microblogs meeting our inclusion criteria by month and Zika virus outbreaks in Asia and by source (e.g., government agency, individual, or other). We identified common misleading or inaccurate content authored by individual micro-bloggers (i.e., not supported by available scientific evidence) through a qualitative review. We used this information to develop and disseminate health awareness material about the Zika virus through China CDC’s 12320 Health Hotline Weibo account. An online survey was conducted to obtain feedback on the material.

**Results:**

We captured 15,888 microblogs meeting our inclusion criteria. Zika-related microblogs peaked in September 2016, corresponding to news reports about the Zika outbreak in Singapore (August to November 2016). Most microblogs (12,994 [82%]) were authored by individual users, followed by media agencies (842 [5%]), businesses (829 [5%]), international organizations (370 [2%]), and Chinese government agencies (235 [1%]). Relevant microblogs primarily focused on clinical symptoms and health risks, modes of transmission, and actions taken by individuals to prevent infection and seek health care. Incorrect and/or mis-leading information from individual users concentrated on modes of transmission and possible treatments. The microblog "#Zika is that far and this close" health campaign was posted on Sina-Weibo and Baidu (Internet search engine in China) on September 18, 2016. Younger respondents (p-value = 0.01), and those with at least a college education (p-value = 0.03), were more likely than other respondents to consider the online campaign reliable and trustworthy.

**Conclusion:**

Routine review of Sina-Weibo and other social media platforms could enhance the ability of public health staff to effectively respond to community concerns and awareness during public health emergencies. Advancements of social media monitoring tools and staff training could help to promote health awareness during emergencies by directly addressing public perceptions and concerns. Various approaches may be needed to reach different at-risk populations, particularly older and less educated populations who may prefer more traditional modes of communication.

## Introduction

On 1 February 2016, the World Health Organization declared Zika virus transmission a public health emergency of international concern [1] due to confirmed links with microcephaly [2, 3] and Guillain-Barré syndrome (GBS) [4]. By early 2016, 20 countries were reporting Zika virus infections [5]. The first case of Zika virus was confirmed and reported in China in February 2016 in a traveler from Venezuela [6]. This was followed by two Zika case-reports from Hong Kong in August 2016 and an outbreak of Zika virus in Singapore from August to November 2016, resulting in 855 cases [7]. As in other newly effected countries, the population in China likely had little to no knowledge or awareness about the Zika virus. Rapid dissemination of scientifically correct and up-to date information was a priority for public health officials in China. Social media platforms could provide a means to understand and effectively respond to community perceptions and concerns about the Zika virus.

The China 12320 Health Hotline Management Center is responsible for receiving and responding to health questions from the public [8]. The Center supports call-centers in each of the 31 provinces and autonomous regions in mainland China and a Weibo social media account. Weibo is a microblogging service in China that allows users to post and share short messages, and attach links, images, or videos to the messages [9–11]. The service had more than 392 million monthly active users in 2017 [12]. Many health agencies in China also maintain Weibo accounts, including the World Health Organization, the Chinese Ministry of Health, and provincial level governments. Microbloggers can use Sina-Weibo to discuss various social issues, environmental topics, and health concerns. Responding to public health emergencies relies on obtaining real-time information from the public to design useful and effective health messaging [13]. This includes identifying and addressing possible knowledge gaps and misinformation related to the public health emergency–information commonly shared through social media. Although surveys and telephone polls can be helpful [14, 15], these are often time consuming or may be not capture community sentiment or misinformation as an outbreak or pandemic evolves over time [16]. Previous analyses of Weibo content have been used to assess perceptions in China about the 2012 Middle East Respiratory Syndrome Coronavirus outbreak [17] and the 2013 avian influenza A(H7N9) outbreak in China [17–20].

Here, we describe a pilot project using information captured from the review and analysis of micro-blogs posted on Sina-Weibo, China’s largest social media platform to develop and disseminate a Zika virus education campaign. We describe our findings and lessons learned, including challenges in rapidly reviewing Zika-related microblogs and considerations for educating the public and promoting healthy behaviors during an evolving pandemic. Findings from this pilot project can be used to develop a more efficient approach to ensure rapid evaluation and dissemination of evidence-based responses that address specific public perceptions and concerns during future public health emergencies.

## Methods

### Social media review and analysis

#### Capturing microblogs from Sina-Weibo

Sina-Weibo is a popular social media platform in China. Individuals, businesses, academic centers, government agencies and international organizations can share text, pictures, and videos with others using mobile devices, tablets and computers. On January 28, 2016, the word limit of Sina-Weibo microblogs changed from a maximum of 140 words to a maximum of 2,000 words. The microblogs captured for this project were posted after February 1, 2016. Approximately 65% of these microblogs were < 100 characters; the remaining 35% were 100 words or more. Longer microblogs were typically posted by official agencies. The platform allows for close to real-time interaction between users. Although the platform is available globally, the majority of users are located in mainland China, primarily among the younger and urban populations in the country. As of December 2017, 392 million persons and organizations were active monthly users, approximately 30% of China’s total population [12].

The microblog search and cleaning were conducted by a professional company using CYYUN Voice Express’ Weibo Spider tool, a cloud calculating Software-as-a-Service (SAAS) platform. The company focuses on internet, artificial intelligence, technology and communication research and has previous experience in performing public health projects (http://www.cyyun.com/index.htm). The Voice Express’ Webio Spider tool captures microblogs by using a combination of RSS feeds, proprietary crawlers, and API access and adheres to the terms of use of Sina-Weibo. For this project, we captured microblog content about Zika virus retrospectively from February 1 to December 31, 2016, and prospectively from June 1 to November 15, 2017. For both time periods, we used the search terms "Zhaika" OR "Zika" OR "Zikv". Although we pilot tested the use of other keywords (e.g., sika and zaika), we relied on these general disease terms (in both Chinese and English) to maximize our search results. Only microblogs set as public were captured and microblogs unrelated to the health topics (e.g., personal emotions and advertisements) were excluded (**S1 File**). For the remaining microblogs, user information, geographical origin, authentication status (i.e., author type such as government agency or individual from Sina-Weibo registration information), number of fans, number of comments, forwarding frequencies, number of likes as well as microblog posting date and microblog content were downloaded into an Excel spreadsheet for further review.

#### Identifying and analyzing relevant microblogs

A single project staff member manually reviewed all captured microblogs to identify those meeting our inclusion criteria. A second staff member reviewed the blogs to ensure agreement and discrepancies were discussed with a third member; inclusion of a captured microblog required agreement by two of three project members. Our inclusion criteria required that a microblog addressed Zika virus, symptoms, transmission risk factors, prevention and control measures, Zika case-reports and outbreaks, or travel warnings or alerts. Blogs not meeting these criteria were excluded, resulting in a final set of microblogs for our analysis.

We described the temporal trends of microblogs meeting our inclusion criteria by month and assessed changes in the frequency of microblog postings in relation to Zika-related events in China as well as elsewhere in Asia. We also described the sources—or authors–of the microblogs as individual users, business, government agencies, international health organizations, and traditional media. These sources were identified according to the authentication status in the user’s profile and, along with place of residence, is included as part of the Sina-Weibo registration process. This information downloaded for each microblog captured by Voice Express’ Webio Spider tool. Each source was further categorized by the number of followers.

We categorized the relevant microblogs into four general categories using a top-down approach [21] based on primary themes identified from social media captured during previous public health emergencies [1, 22–26]. For this pilot project, we reviewed and placed each microblog into one of four theme categories: 1) Zika-related action taken by individuals, 2) clinical information about the health impact of the Zika virus and modes of transmission, 3) Zika case and outbreak reports, and 4) global and country-specific control and prevention measures. This approach allowed us to more rapidly translate our review findings into a Zika virus awareness education campaign–including addressing public perceptions in each of these major categories. Each microblog could only be placed in a single theme; agreement among three project staff was required for placement of microblogs with potentially complex content. We described these themes by source (e.g., individuals or government agencies, international organization) and year (2016 and 2017).

We further identified microblogs authored by individual users that included incorrect or misleading information about the Zika virus. If any part of the microblog was inaccurate or misleading, including embedded links, the microblog was categorized as inaccurate or misleading. We defined incorrect or misleading content as a claim or information that was false or inaccurate according to available scientific evidence [27]. Content accuracy was verified by information published by WHO, China CDC, and US CDC. If necessary, a third staff member, a subject matter expect, was included in the discussion to assist in categorizing the microblog.

We focused microblogs authored by individual users as a proxy for likely knowledge gaps in the general population.

### Online Zika educational campaign

#### Content and format

We developed an online educational campaign to address the inaccurate and misleading information identified from the review and analysis of microblogs posted by individual users during 2016 across the four major theme categories. The content addressed the following topics: Zika virus cannot be cured (treatment), Zika is not transmitted by mosquitoes (transmission), Have no idea about the symptoms of Zika (clinical presentation), Have no idea about the harm of Zika and its protective measures (risk of Zika virus and effective prevention measures), Zika has no harm to pregnant women. (risk of Zika virus). The goal of the campaign was to inform appropriate public actions and reactions to the Zika virus [13], including promoting healthy behavior to prevent Zika virus transmission, particularly among pregnant women. The material included a combination of cartoons and limited text. Use of images, including cartoons, is recommended by the government department as part of the “Healthy China Action” initiative [28], to better educate diverse populations with varying levels of health literacy [28].

#### Pilot testing and distribution of the Zika education material

The education campaign material was pilot tested in two focus groups of 16 persons each to ensure comprehensibility and effectiveness among persons with varying literacy levels. Focus group members were recruited as volunteers from staff and family members of the National Health Hotline Management Center. Volunteers included persons with different professional and educational backgrounds (e.g., intern, security guard, professional staff). The two 60-minute focus group sessions were coordinated and led by Health Hotline project staff on 1 and 7 September 2017. Participants were provided the chance to review and address questions about the comprehensibility, usefulness, and overall impressions of the campaign material. Feedback from the focus group participants (e.g., improve images and information about Zika virus infection in pregnant women, and remove unnecessary technical verbiage) was incorporated into the materials. Final Zika campaign materials were distributed through National Health Hotline 12320’s official Sina-Weibo account from September 18 to September 24, 2017. The education materials were also shared on the National Health Hotline’s website. Other Provincial Health Hotline offices could also share the material via provincial supported Sina-Webio and website.

### Evaluation of education campaign

To evaluate the effectiveness of the campaign, we developed and shared a survey to followers of the Health Hotline’s Sina-Weibo site to obtain feedback on the Zika virus campaign materials. All persons greater than 18 years of age and able to read Chinese were able to response to the survey. The survey requested demographic information, frequency of visiting the Health Hotline’s microblog, preferred source of receiving health related information, and basic knowledge and awareness about Zika virus disease. A copy of the survey is available (in English and Chinese) in **S2 File**. The online survey was programmed using Tool called Questionnaire Star and posted on the Health Hotline’s Sina-Weibo account from September 18 to September 24, 2017. Respondents represented a convenience sample of Sina-Weibo users; no sample size or power calculations were generated. Responses were automatically saved on a served at China CDC. We downloaded all responses from the online questionnaire provided during this time period into Excel for cleaning and into SPSS (version 23) for analysis. We described feedback on the Zika education material by age and education level.

The China Centers for Disease Control and Prevention approved the project as a program activity. The project was also approved United States Centers for Disease Control and Prevention’s Human Subjects Research Office (HSRO) as a non-research program evaluation project. All personal identifying information captured during the microblog search were removed prior to analysis. Sina-Weibo users provided consent to the campaign evaluation questionnaire by agreeing to have their responses reviewed and analyzed as part of this project.

### Ethic approval and consent to participate

China CDC approved this project as a program activity, and therefore, the protocol was exempt from review by the ethical review board. The project was also reviewed in accordance with CDC human research protection procedures and was determined to not constitute human-subjects research as defined in 45CFR46.102(l). All Sina-Weibo microblogs included in this analysis were publicly available. No personal identifying information was included in the analysis nor in the reports and manuscripts generated from the project.

## Results

### Social media review and analysis

Between February 1 and December 31, 2016 and June 1 to November 31, 2017, a total of 24,150 social media microblogs were captured using our search criteria. Of these, 8,262 (34%) microblogs were excluded as irrelevant (primarily related to commercial advertisements, housing information, as well as financial information and political commentary). The remaining 15,888 Zika-related microblogs were associated with 14,950 comments, 2,987 likes, 2,877 replies, and were forwarded 2,555 times. A mean of 1,239 Zika-related microblogs were authored each month in 2016, peaking in September with 8,700 microblogs (**S1 Fig**). The September peak corresponds to news report about two Zika cases detected in Hong Kong (August 2016) and the Zika outbreak in Singapore (August to November 2016). Between June 1 to November 31, 2017, a mean of 110 Zika-related microblogs were authored each month.

Of the 15,888 microblogs included in our analysis, most (12,994 [82%]) were authored by individual users, followed by media agencies (842 [5%]), businesses (829 [5%]), and international organizations (370 [2%]). Additional microblogs were authored by Chinese government agencies (235 [1%]), academic institutions (51 [< 1%]), and other groups such as social and campus organizations (567 [4%]) (**S1 Table**). The 15,888 microblogs captured in our project were authored by 2,108 unique authors. Of these unique authors, 663 (31%), including 422 individual users, had more than 100,000 followers (**S3 File**).

The 15,888 microblogs included in our analysis were categorized into the following themes: Zika-related actions taken by individuals (7,650 [48%]), information about modes of transmission and clinical symptoms and health risks (3,741 [24%]), Zika case and outbreak updates (2,551 [16%]), and information on global and country-specific control and prevention measures (1,945 [12%]) (**S2 Table**). Microblog themes varied by author source. The majority (10,463 [81%]) of microblogs authored by individual users addressed Zika virus symptoms, risks for pregnant women, and modes of transmission as well as actions taken by individuals to prevent infection or seek care for possible infection, whereas the majority (160 [72%]) of microblogs authored by government offices addressed Zika virus case and outbreak reports, and global and country level outbreak response and control measures.

Of the 12,994 microblogs authored by individual users, 100 (< 1%) were classified as inaccurate or misleading”. For example, one blogger stated that “Zika virus and other nervous system diseases are the consequences of genetic modification”, while another indicated that “Zika virus is a new virus invented by people from different countries”. The 100 microblogs including misleading or incorrect content were created by 96 unique individual users and were forwarded 15,423 times. Of the 96 unique individual users, 5 (5%) had more than 100,000 followers.

### Online campaign and evaluation

Based on findings from our social media analysis, the National Health Hotline 12320 management office developed and released the microblog post "#Zika is that far and this close" on September 18, 2016. The microblog included illustrations and graphics to communicate knowledge and awareness about Zika virus, transmission, and prevention (**S2 Fig**). The resulting education material included five cartoon panels. These included: Epidemic situation, Basic knowledge of Zika virus disease, How to prevent Zika virus disease in pregnant women, How can the public prevent it, Travel related issues to Zika virus disease.

The microblog was read and forwarded 288,000 and 427 times, respectively.

In the follow-up evaluation, 157 (98%) of 161 respondents stated that the blog was well designed, and 143 (89%) forwarded and commented on the post (**S3 Table**). Younger respondents (p-value = 0.01), and those with at least a college education (p-value = 0.03), were more likely to consider the online campaign as reliable and trustworthy compared to other respondents.

## Discussion

By reviewing and analyzing more than 15,000 Sina-Weibo posts, we assessed public awareness and concerns about Zika virus transmission during 2016 and for six-months during 2017. Information was captured from microblogs authored by individual users by media organizations, businesses, and government agencies. Misleading and incorrect information was identified among individual microbloggers and most commonly involved false or inaccurate content about the source of the Zika virus and possible treatments. We used these findings to develop and disseminate an online education campaign using illustrations and graphics. The review and analysis of the Zika related microblogs allowed us to respond with accurate information specifically targeting identified public concerns and misconceptions.

### Review and analysis of Sina-Weibo microblogs

Although Zika virus had been circulating in the Americas since late 2015, little information about Zika was shared through social media in China until May 2016. Microblog content about Zika later peaked in September, corresponding with news reports about the Zika outbreak in Singapore [7]. The spread and high harmfulness of public health emergencies are often easy to cause panic among the public and lead to collective discussion. As an important carrier of information generation and dissemination, social media platform plays an indispensable role. If the relevant departments can not eliminate the extreme views and negative emotions in social media in time, it is very likely to lead to the violation and extreme behavior of the masses.

Interestingly, the social media reaction in China appeared to be greater to the outbreak in Singapore than from individual reports of imported Zika cases in Guangdong Province. This finding could reflect the impact of information from Chinese residents in Singapore and fears about widespread Zika virus transmission, or limited concerns about sustained transmission following the imported cases into Guangdong Province, or both. Few Zika related blogs were posted in late 2016 and during the project period in 2017. This decrease could suggest the impact of seasonal changes and less concern about mosquito-borne diseases, including Zika-virus, or possibly public fatigue. Similar findings were observed in the analysis of Weibo microblogs created during the influenza H7N9 outbreak [13]. Microblogs about the outbreak increased a few days after reports of the first three human cases, peaking after government reports about a H7N9 case in an infant in Beijing, and then quickly declining thereafter [13]. Because we disseminated the Health Hotline campaign in September 18, 2016, we were unable to directly address the impact of this campaign–or in general knowledge and awareness—on Weibo user’s knowledge and perceptions following the peak of the outbreak in Asia.

During 2016, Zika related social media content varied substantial by source. Although government agencies disseminated 1,389 microblogs during 2016, these blogs included scientific text and, according to public comments, were difficult to understand. Additionally, Sina-Weibo postings by government agencies (e.g., Healthy China, the official Weibo account of the National Health Commission), National Health Hotline 12320, Beijing CDC and Beijing Health Hotline 12320) represented 1% of all Zika-related microblogs captured during this project. Increasing the frequency of microblogs from these Chinese health authorities in timely and accurate and easy to understand messages could help minimize public miss-conceptions and concerns, particularly after the initial Zika cases among travelers reported from Guangdong Province in February 2016. Additional efforts to ensure that content of government microblogs is consistent across both national and provincial level agencies and provides the most up-to-date and accurate information and easily understand manner (e.g., cartoons)–could further increase trust.

### Education campaigns during public health emergencies

Although more than 500 million Chinese access Sina-Weibo daily, information described in this analysis is likely unrepresentative of the general population in China. About 5% of Weibo users contribute to more than 80% of original micro-blogs, more than half of Weibo subscribers have never created a micro-blog [26]. Because online access is highest in urban areas among the younger population [27], concerns and perceptions of rural and older populations may have not been reflected in our findings. Interestingly, the young and educated were also most likely to consider the online education campaign as credible and useful. We conducted our initial social media search retrospectively in late 2016 and prospectively for six months in 2017. We were unable to develop and disseminate information during the peak period of Zika transmission in Asia. This limited the usefulness of our project as well as our ability to assess the impact of our campaign on Zika related public knowledge and perceptions. Sharing the latest scientific information and official policies in text format is unlikely to engage the public’s interest [13].

### Routine monitoring and evaluation

Monitoring and analyzing social media are increasingly important components of public health practice. On the social media platform, some unscrupulous media or individuals by publishing untrue reports and false information, to create gimmicks and guide public opinion, resulting in the flooding of various irrational emotions, negative emotions and even extreme emotions in the network environment. Public opinion information of public health emergencies spreads along with users’ forwarding and commenting behavior. Users, as information recipients, are not only responsible for public health emergencies, but also responsible for public health emergencies, The change of their attitude and behavior is influenced by information publishers and information content. The ability to monitor public perception and concerns of an evolving outbreak allows public health agencies to effectively address community concern and prevent further transmission [20, 21]. Social media can also provide a quantitative measure of public attention toward a specific disease or outbreak [22, 23]. For example, although the majority of social media included in our analysis corresponded to outbreaks and case reports occurring outside of China, this finding signaled a public concern and need for additional information for all Sina-Weibo users. Additionally, we captured microblogs authored by individual users communicating misleading or incorrect information about the origins of the Zika virus and possible treatment options.

Additionally, monitoring social media can be much more efficient and cost-effective than traditional telephone-polls and household surveys for assessing community awareness and concerns of an evolving public health emergency [24]. Next steps should include further social network analyses to understand the role of networks in the spread of online information (including misinformation or conspiracies [25]) and the development of automated approaches to facilitate social media content analysis. On the other hand, in public health emergencies, social media shows important social value with its advantages of wide audience, fast communication, openness and freedom. The facts need to know and awareness of protection of public’s make them put forward higher requirements on the source and accuracy of health information. These two factors will affect the value of information to a certain extent. Although social media highlights new vitality, how to promote effective sharing of information in public emergencies still needs further discussion. The reliability and accuracy of information released by the government have a certain guiding role for public opinion.

## Conclusion

Public health emergencies are different from general events, which have the characteristics of suddenness, diversity of causes, impact on groups and severity of harm. Social media is a platform for content production and sharing based on user relationship on the Internet. Users can share opinions, knowledge and experience in social media. Social media can provide information about public knowledge and awareness as well as possible misinformation about emerging or re-emerging infectious diseases, such as Zika virus, in near-real time. In this project, we were able to capture content about Zika from over 10,000 microblogs from Sina-Weibo and developed an online education campaign using illustrations and graphics to address identified misleading information and public concerns. Public health emergencies may cause serious damage to public health, resulting in the change of people’s health beliefs and attitudes. When people get health information through authoritative media, they will have a clear understanding of the susceptibility and severity of the disease. Access to and use of such information can help other users in social media avoid risks, so people tend to share information [29]. In the future, this effort can be implemented earlier during a potential public health emergency to help ensure the public has correct and accurate information. Additionally, public health officials can work to ensure that all national and local government agencies, as well as leading public opinion leaders, communicate consistent and easy to understand messages across various platforms (WeChat and Sina-Weibo). This could help improve public confidence and guarantee that different segments of the population access the same evidence-based information. In addition, the government’s official media should make authoritative interpretation of the hazards and prevention measures of emergencies, so as to promote the formation of citizens’ health belief identity; Social media platform can make full use of big data to monitor and intelligently analyze public opinion and guide public opinion.

## Supporting information

S1 Fig Zika virus-related microblogs posted on Weibo from February1-December 31, 2016 and June 1-November 30, 2017. Microblogs identified using CYYUN Voice Express Weibo Spider tool and relevant inclusion criteria (n = 15,888).
(DOCX)

S2 Fig Zika virus educational cartoon, posted on the 12320 Health Hotline’s Weibo account on September 20, 2016 (Original Chinese version version). (DOCX)

S1 Table Zika-related microblogs posted on Weibo from February1-December 31, 2016 and June 1-November 30, 2017, by author source. Microblogs identified using CYYUN Voice Express Weibo Spider tool.
(DOCX)

S2 Table Main content themes of Zika-related microblogs posted on Weibo from February1-December 31, 2016 and June 1-November 30, 2017, by author type. Microblogs identified using CYYUN Voice Express Weibo Spider tool and relevant inclusion criteria.
(DOCX)

S3 Table Survey responses on effectiveness of online Zika education campaign shared via the 12320 Health Hotline’s Management office’s Weibo and Baidu accounts from September 14–28, 2016. (DOCX)

S1 File List of Exclusion Criteria. (DOC)

S2 File Zika Education Campaign Evaluation Questionnaire posted on the Health Hotline’s Sina-Weibo account from September 18 to September 24. All partial and completed responses were downloaded and analyzed.
(DOC)

S3 File Unique authors posting Zika-related microblogs from February 1-December 31, 2016 and June 1-November 30, 2017, China, by number of followers. (DOC)

## Abbreviations

China CDC: China Center for Disease Control and Prevention
US CDC: United States Centers for Disease Control and Prevention
