# Supplementary material for: Using Sina-Weibo microblogs to inform the development and dissemination of health awareness material about Zika virus transmission, China, 2016–17
Source: PLoS One. 2022 Jan 27;17(1):e0261602. doi: 10.1371/journal.pone.0261602 (PMC8794198; doi:10.1371/journal.pone.0261602)
Supplement: S1 Fig — Microblogs identified using CYYUN Voice Express Weibo Spider tool and relevant inclusion criteria (n = 15,888). (DOCX) [file pone.0261602.s001.docx]

**Figure 1.** Zika virus-related microblogs posted on Weibo from February1-December 31, 2016 and June 1-November 30, 2017. Microblogs identified using CYYUN Voice Express Weibo Spider tool and relevant inclusion criteria (n = 15,888)*.


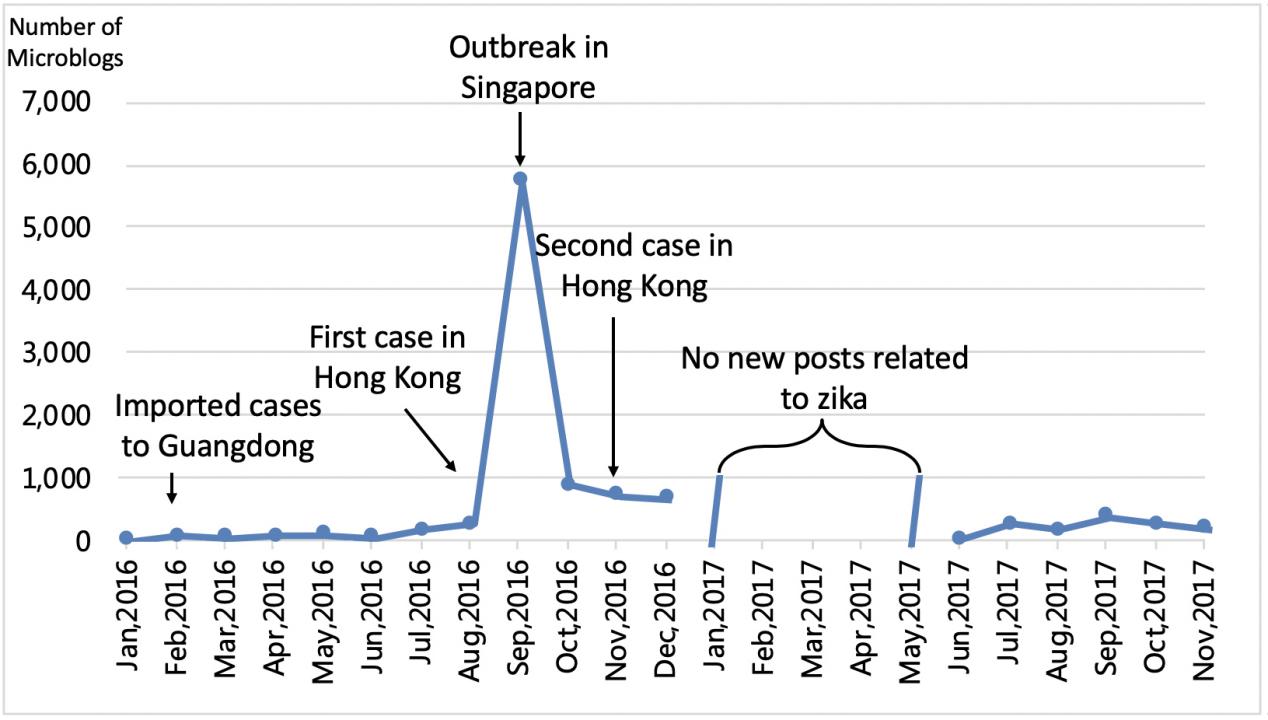


*The World Health Organization declared Public Health Emergency of International Concern on 1 February 2016.

Imported Zika virus cases were initial detected in Guangdong Province among Chinese nationals returning home from work-related travel to Venezuela. Singapore Zika virus outbreak resulted in 855 cases. No Zika-related microblogs were identified using search terms and inclusion criteria from February to June 2017.
