# Supplementary material for: Using Sina-Weibo microblogs to inform the development and dissemination of health awareness material about Zika virus transmission, China, 2016–17
Source: PLoS One. 2022 Jan 27;17(1):e0261602. doi: 10.1371/journal.pone.0261602 (PMC8794198; doi:10.1371/journal.pone.0261602)
Supplement: S2 Fig — (DOCX) [file pone.0261602.s002.docx]

**Figure 2.** Zika virus educational cartoon, posted on the 12320 Health Hotline’s Weibo account on September 20, 2016 (Original Chinese version version)*.

**
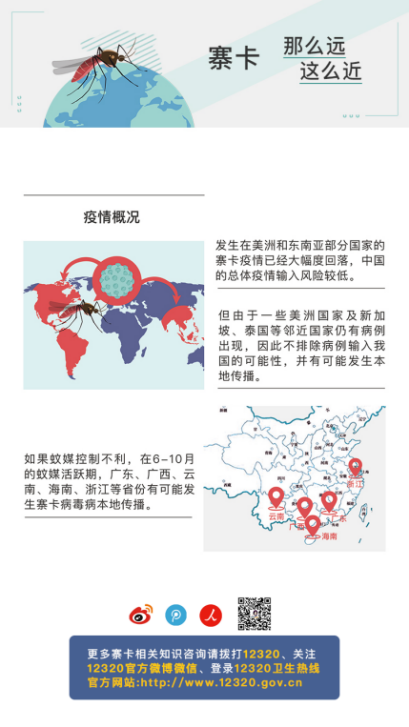

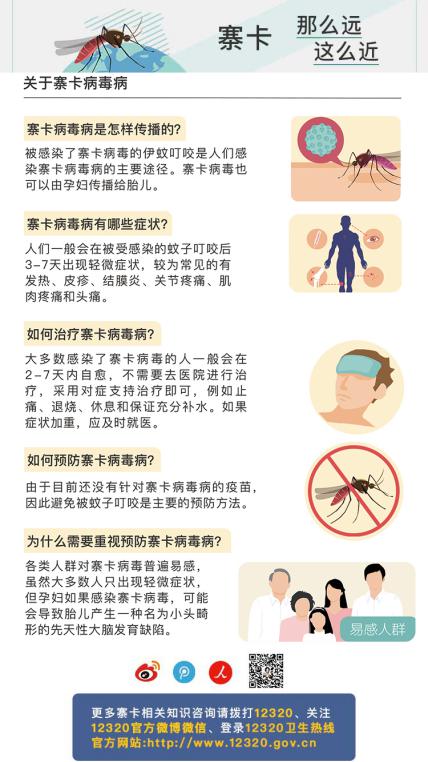

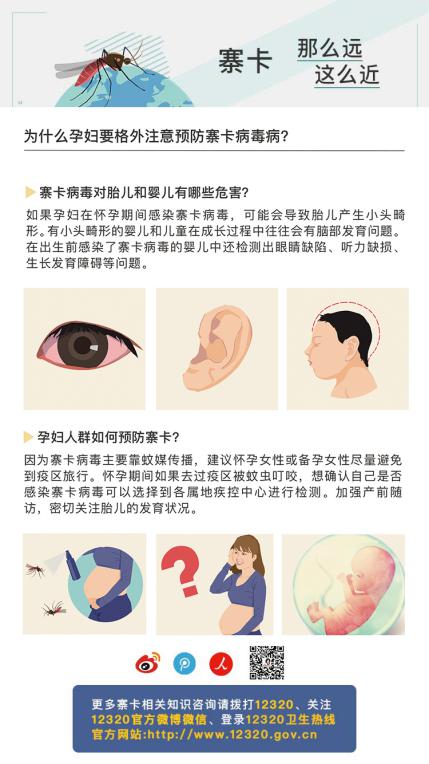
**

**
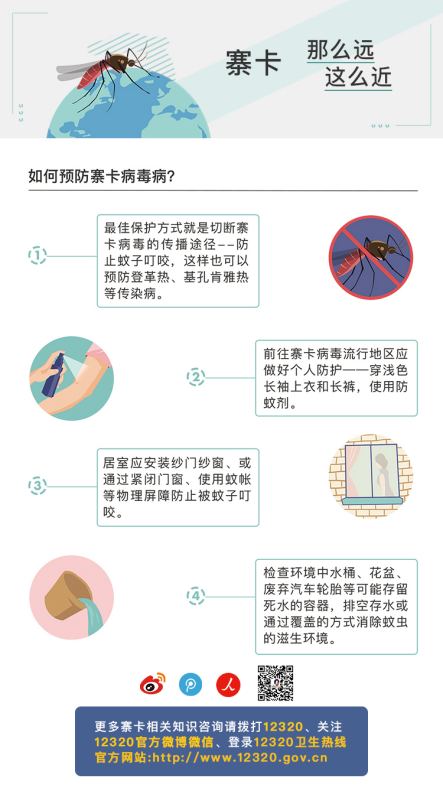

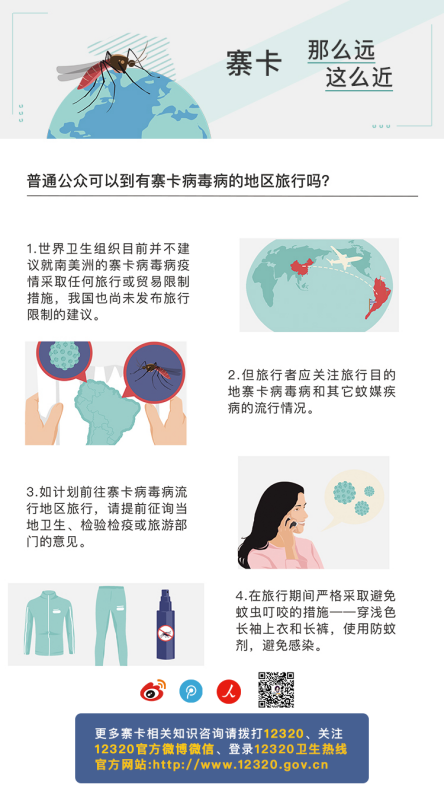
**

*Developed by Health Hotline project staff as part of this pilot project.
