# Supplementary material for: Using Sina-Weibo microblogs to inform the development and dissemination of health awareness material about Zika virus transmission, China, 2016–17
Source: PLoS One. 2022 Jan 27;17(1):e0261602. doi: 10.1371/journal.pone.0261602 (PMC8794198; doi:10.1371/journal.pone.0261602)
Supplement: S1 Table — Microblogs identified using CYYUN Voice Express Weibo Spider tool. (DOCX) [file pone.0261602.s003.docx]

**Table 1.** Zika-related microblogs posted on Weibo from February1-December 31, 2016 and June 1-November 30, 2017, by author source. Microblogs identified using CYYUN Voice Express Weibo Spider tool*.

| **Author source** | **Year – N (%)** | | | | | |  |
| --- | --- | --- | --- | --- | --- | --- | --- |
|  | **2016** | | **2017** | | **Total** | |  |
| **Individual Users** | 12,189 (82) |  | 805 (75) |  | 12,994 (82) |  | |
| **Media Agencies** | 777 (5) |  | 65 (6) |  | 842 (5) |  | |
| **Businesses** | 775 (5) |  | 54 (5) |  | 829 (5) |  | |
| **International Organizations** | 319 (2) |  | 51 (5) |  | 370 (2) |  | |
| **Government Offices** | 175 (1) |  | 60 (5) |  | 235 (1) |  | |
| **Academic Institutions** | 47 (< 1) |  | 4 (< 1) |  | 51 (< 1) |  | |
| **Other**** | 532 (4) |  | 35 (3) |  | 567 (4) |  | |
| **Total** | 14,815 (100) |  | 1,073 (100) |  | 15,888 (100) |  | |

*Total excludes 8,262 (34%) unrelated microblogs (primarily related to political commentary, commercial advertisements, housing information, and financial issues).

**Other includes microblogs from various campus and social organizations.
