# Supplementary material for: Using Sina-Weibo microblogs to inform the development and dissemination of health awareness material about Zika virus transmission, China, 2016–17
Source: PLoS One. 2022 Jan 27;17(1):e0261602. doi: 10.1371/journal.pone.0261602 (PMC8794198; doi:10.1371/journal.pone.0261602)
Supplement: S2 Table — Microblogs identified using CYYUN Voice Express Weibo Spider tool and relevant inclusion criteria. (DOCX) [file pone.0261602.s004.docx]

**Table 2.** Main content themes of Zika-related microblogs posted on Weibo from February1-December 31, 2016 and June 1-November 30, 2017, by author type*. Microblogs identified using CYYUN Voice Express Weibo Spider tool and relevant inclusion criteria.

|  | **Microblog Content Themes** | | | | | | | |  | | | |
| --- | --- | --- | --- | --- | --- | --- | --- | --- | --- | --- | --- | --- |
|  | **2016 - N (%)** | | | | **2017 - N (%)** | | | | **Total** | | |  |
| **Source** | **Clinical information-health risks, and modes of transmission**** | **Reports on Zika virus cases and outbreaks**** | **Global and country-level outbreak response and control measures**** | **Actions taken by individuals in response to Zika**** | **Clinical information-health risks, and modes of transmission** | **Reports on Zika virus cases and outbreaks** | **Global and country-level outbreak response and control measures** | **Actions taken by individuals in response to Zika** | **All** | |  |  |
| **Individual Users** | 2,460 (75) | 1401 (66) | 707(40) | 7620 (100) | 353 (78) | 350 (83) | 72 (43) | 30 (100) | 12,993(82) |  |  |  |
| **International Organizations** | 121 (4) | 96 (5) | 102 (6) | 0 | 16 (4) | 21(5) | 13 (8) | 0 | 369 (2) | |  |  |
| **Media Agencies** | 170 (5) | 202 (9) | 405 (23) | 0 | 35 (8) | 11(3) | 19 (11) | 0 | 842 (5) | |  |  |
| **Government Offices** | 54 (2) | 41(2) | 81(5) | 0 | 21 (5) | 17(4) | 21 (13) | 0 | 235 (1) | |  |  |
| **Academic Institutions** | 14 (< 1) | 11 (1) | 22 (1) | 0 | 2 (< 1) | 1 (< 1) | 1 (1) | 0 | 51 (< 1) | |  |  |
| **Businesses** | 238 (7) | 181 (8) | 357 (20) | 0 | 19 (4) | 16 (4) | 19 (11) | 0 | 830 (5) | |  |  |
| **Other***** | 230 (7) | 199 (9) | 104 (6) | 0 | 8 (2) | 5 (1) | 22 (13) | 0 | 568 (3) | |  |  |
| **Total** | 3,287 | 2,131 | 1778 | 7,620 (100) | 454 | 421 | 167 | 30 (100) | 15,888 (100) | |  |  |

*Percentages are estimated by column (i.e., distribution of author source within each microblog theme).

** Definitions for each theme：

Clinical information-health risks, and modes of transmission：Symptoms of Zika infection and ways of transmission

Reports on Zika virus cases and outbreaks：The number of Zika cases and the progress of the epidemic situation

Global and country-level outbreak response and control measures：Global strategy for Zika epidemic

Actions taken by individuals in response to Zika：Personal practices and actions in the face of epidemic situation

*** Other includes microblogs from various campus and social organizations.
