# Supplementary material for: Using Sina-Weibo microblogs to inform the development and dissemination of health awareness material about Zika virus transmission, China, 2016–17
Source: PLoS One. 2022 Jan 27;17(1):e0261602. doi: 10.1371/journal.pone.0261602 (PMC8794198; doi:10.1371/journal.pone.0261602)
Supplement: S3 Table — (DOCX) [file pone.0261602.s005.docx]

**Table 3**. Survey responses on effectiveness of online Zika education campaign shared via the 12320 Health Hotline’s Management office’s Weibo and Baidu* accounts from September 14-28, 2016.

|  | **No.** | **Viewed**  **Material**  **N (%)** | **Forwarded or commented**  **on material**  **N (%)** | **Consider material reliable**  **N (%)** |
| --- | --- | --- | --- | --- |
| **Age (years)** |  |  |  |  |
| <30 | 69 | 52 (75.4) | 44 (63.8) | 52 (75.4) |
| 31-40 | 47 | 34 (72.3) | 30 (63.8) | 34 (72.3) |
| 41-50 | 22 | 15 (68.2) | 13 (59.1) | 15 (68.2) |
| >51 | 23 | 8 (34.8) | 8 (34.8) | 10 (43.5) |
| **Gender** |  |  |  |  |
| Male | 89 | 52 (58.4) | 44 (49.4) | 53 (59.6) |
| Female | 72 | 57 (79.2) | 51 (70.8) | 58 (80.6) |
| **Education** |  |  |  |  |
| < High school | 20 | 5 (25.0) | 4 (20.0) | 5 (25.0) |
| High school | 17 | 9 (52.9) | 6 (35.3) | 9 (52.9) |
| Junior college | 27 | 16 (59.3) | 13 (48.2) | 18 (66.7) |
| >Junior college | 97 | 79 (81.4) | 72 (74.2) | 79 (81.4) |

*Baidu is the major internet search engine used in China.
