# Supplementary material for: Using Sina-Weibo microblogs to inform the development and dissemination of health awareness material about Zika virus transmission, China, 2016–17
Source: PLoS One. 2022 Jan 27;17(1):e0261602. doi: 10.1371/journal.pone.0261602 (PMC8794198; doi:10.1371/journal.pone.0261602)
Supplement: S1 File — (DOC) [file pone.0261602.s006.doc]

**Supplement 1 – List of Exclusion Criteria**

The Sina-Weibo search terms included the terms "Zhaika", "Zka", "Zika", "Zikv". Microblogs referring the following topics were excluded (n=8,262):

- Personal emotions unrelated to zika
- News voting unrelated to zika
- Personnel report unrelated to zika
- RMB exchange unrelated to zika.
- Implanted advertisements unrelated to zika.
- Lottery purchasing and propaganda unrelated to zika
- Entertainment and game information unrelated to zika
- Housing leasing and sale information unrelated to zika
- Job-hunting information unrelated to zika
- Home service information unrelated to zika
- Auto sales, leasing and maintenance information unrelated to zika
- Financial lending and loan information unrelated to zika
- Stock information unrelated to zika
- Clothing sales, hairdressing, cosmetics and makeup information unrelated to zika (including but not limited to the above information)

Same exclusion criteria were applied to all 24,150 microblogs captured during the retrospective (February 1 to December 31, 2016) and prospective (June 1 to November 31, 2017) search.
