# Supplementary material for: Using Sina-Weibo microblogs to inform the development and dissemination of health awareness material about Zika virus transmission, China, 2016–17
Source: PLoS One. 2022 Jan 27;17(1):e0261602. doi: 10.1371/journal.pone.0261602 (PMC8794198; doi:10.1371/journal.pone.0261602)
Supplement: S2 File — All partial and completed responses were downloaded and analyzed. (DOC) [file pone.0261602.s007.doc]

**Supplement 2 - Zika Education Campaign Evaluation Questionnaire posted on the Health Hotline’s Sina-Weibo account from September 18 to September 24. All partial and completed responses were downloaded and analyzed.**

1. **English version**

**Questionnaire on the Dissemination Effect of theCartoon of Zika Virus Disease**

Hello, @National health 12320 official Weibo platform launches this questionnaire in order to understand your opinion & attitude of this health dissemination activity, and also the demand for Zika Virus Disease related information. We hope to get your cooperation. We promise that all the content you will answer will be kept confidential and only for our data analysis. Thanks for your participation. We also hope that you can give us valuable advice.

I Basic information

1. Age:__________

2. Gender: ( ) 1) male 2) female

3. Degree of education: ( )

1) Primary school or below

2) Junior middle school

3) Senior middle school or Technical secondary school

4) Junior college

5) Bachelor's degree

6) Master degree or above

4. Occupation: ( )

1) Civil servant

2) Administrative personnel of public institutions

3) Enterprise staff

4) Business services staff

5) Medical and health personnel 6) Educator

7) Transportation personnel

8) Temporary worker and unemployed person

9) Business manager, self-employed business owner and self-

employed entrepreneur

10) Retired person 11) Student

12) Domestic worker 13) Other

5. Personal monthly income: ( )

1) <1500 yuan 2) 1500 yuan ~ 3000 yuan

3) 3001 ~ 5000 yuan

4) 5001 yuan ~ 8000 yuan 5) >8001 yuan

II The attention situation of the health dissemination activity of the Zika Virus on Weibo platform

6. Have you seen the cartoon of Zika Virus disease released by @National health 12320 on September 18-24? ( )

1) Yes 2) No (go to question 12)

7. Do you feel that the information of Zika Virus disease issued by @National health 12320 is reliable? ( )

1) very reliable 2) reliable 3) very untrustworthy

8. Is the information of Zika Virus released by @National health 12320

appealing to you? ( )

1) Yes 2) No

9. Did you forward or comment the information about Zika Virus

disease released by @National health 12320? ( )

1) Yes 2) No

10. Is the information about Zika Virus disease released by @National

health 12320 helpful for you? ( )

1) Very helpful 2) helpful 3) Relatively helpful

4) Not helpful at all (please explain why:_____________________________)

11. What information about Zika Virus disease released by @National

health 12320 is most helpful for you?

1) The transmission pathway of Zika Virus disease

2) The general situation of Zika Virus disease in China

3) The main harm of Zika Virus disease

4) The prevention method of Zika Virus disease

5) other (please specify:________________________________)

III The use of Weibo platform and the attention for Use and

@National health 12320

12. How often do you use Weibo platform? ( )

1) 0-2 times a day 2) 3-5 times a day 3) more than 5 times a day

13. Have you followed and seen the information on the National

health 12320 official weibo platform many times in two continuous

weeks?

1) often 2) once in a while 3) never

14. Do you think the content of @national health 12320 is

authoritative and reliable? ( )

1) very reliable 2) reliable 3) not very reliable 4) not reliable at all

IV The demand of the information related to Zika Virus disease

15. What kind of information would you like to know about the zika

virus? ( You can have multiple choices )

1) Infection source 2) Incubation period 3) Route of transmission

4) Susceptible population 5) Clinical manifestation

6) Prevention measures

7) Other ______________________________________

16. Which way would you like to obtain information about zika

virus disease? ( You can have multiple choices )

1) The official Weibo platform of national health 12320

2) The official Wechat platform of national health 12320

3) SMS message from 12320

4) Official website of 12320

5) Brochure from 12320

6) other_____________________

Thanks for your cooperation. Wish you a happy life, happy work and happy family!

1. **Original version in Chinese**

寨卡病毒病漫画传播效果调查问卷

您好，为了解您对此次健康传播活动的看法和态度，以及对寨卡病毒病相关信息的需求，@全国卫生12320官方微博发起此次调查问卷，希望能得到您的配合。我们承诺您回答的全部内容都将予以保密，只供我们对相关问题进行数据分析。感谢您的参与，也希望您能给我们提出宝贵的意见。

一、基本情况

1．年龄： 岁

2．性别：（ ） 1）男 2）女

3．文化程度：（ ）

1）小学及以下 2）初中 3）高中或中专 4）大专

5）本科 6）硕士及以上

4．职业：（ ）

1）公务员 2）事业单位行政人员

3）企业职工 4）商业服务业人员

5）医疗卫生人员 6）教育工作者

7）交通运输人员 8）临时工及无业人员

9）企业高管及民营企业业主及个体经营者

10）离退休人员 11）学生

12）家务劳动者 13）其他

5．本人月收入在：（ ）

1）<1500元 2）1500元～3000元 3）3001元～5000元

4）5001元～8000元 5）>8001元

二、寨卡病毒病微博健康传播活动关注情况

6. 您看过@全国卫生12320在9月18-24日发布的寨卡病毒病的相关漫画吗？ （ ）

1）看过 2）没看过（跳转至第12题）

7. 您觉得@全国卫生12320发布的寨卡病毒病信息可信度高吗？( )

1）非常可信 2）比较可信 3)非常不可信

8. @全国卫生12320发布的寨卡病毒病信息对您有吸引力吗？( )

1）有 2）没有

9. 您是否对@全国卫生12320发布的寨卡病毒病信息进行了转发和评论？( )

1）是 2）否

10. @全国卫生12320所提供的寨卡病毒病相关信息对您有帮助吗？（ ）

1）很有帮助 2）有较大帮助 3）有一些帮助

4）完全没有帮助（请说明原因： ）

11. @全国卫生12320所提供的哪方面寨卡病毒病相关信息对您最有帮助？

1）寨卡病毒病传播途径 2）寨卡病毒病在中国的总体情况

3）寨卡病毒病的主要危害 4）寨卡病毒病的预防方法

5）其他 （请说明： ）

三、微博使用和关注情况

12．您使用微博的频率是？ （ ）

1）每天0-2次 2）每天3-5次 3）每天5次以上

13．您是否在连续两周时间段内经常关注全国卫生12320的官方微博（ ）

1）经常 2）偶尔 3）从不

14．您认为@全国卫生12320发布的内容是权威、可信任的吗？（ ）

1）非常值得信任 2）比较值得信任

3）不太值得信任 4）不值得信任

三、对寨卡病毒病相关信息的需求情况

15．您最希望了解哪些寨卡病毒病的相关信息（可多选）？（ ）

1）传染源 2）潜伏期 3）传播途径

4）易感人群 5）临床表现 6）预防措施

7）其他

16．您希望通过哪些方式获取寨卡病毒病的相关信息？（可多选）（ ）

1）全国卫生12320官方微博

2）全国卫生12320官方微信

3）12320短信息

4）12320官方网站

5）12320宣传手册

6）其他
