# Supplementary material for: Using Sina-Weibo microblogs to inform the development and dissemination of health awareness material about Zika virus transmission, China, 2016–17
Source: PLoS One. 2022 Jan 27;17(1):e0261602. doi: 10.1371/journal.pone.0261602 (PMC8794198; doi:10.1371/journal.pone.0261602)
Supplement: S3 File — (DOC) [file pone.0261602.s008.doc]

**Supplement 3**. **Unique authors posting Zika-related microblogs from February 1-December 31, 2016 and June 1-November 30, 2017, China, by number of followers.**

| **Number of followers** | **2016-2017** | | | | | | | |
| --- | --- | --- | --- | --- | --- | --- | --- | --- |
|  | **Individual users** | **Media agencies** | **International Organizations** | **Government Offices** | **Academic Institutions** | **Businesses** | **Other*** | **Total** |
| **1 - 1,000** | 1,207 | 0 | 0 | 0 | 1 | 35 | 0 | 1,242 |
| **1,001 - 10,000** | 76 | 0 | 0 | 0 | 1 | 27 | 0 | 105 |
| **10,001 - 100,000** | 19 | 0 | 0 | 0 | 0 | 32 | 47 | 98 |
| **> 100,000** | 422 | 112 | 49 | 31 | 3 | 14 | 32 | 663 |
| **Total** | 1,724 | 112 | 49 | 31 | 5 | 108 | 79 | 2,108 |

*Other includes microblogs form various campus and social organizations.
